# The Old-Age Healthy Dependency Ratio in Europe

**DOI:** 10.1007/s12062-012-9068-6

**Published:** 2012-07-07

**Authors:** Magdalena M. Muszyńska, Roland Rau

**Affiliations:** 1 Institute of Statistics and Demography, Warsaw School of Economics, ul. Madalińskiego 6/8, 02-513 Warsaw, Poland; 2 University of Rostock, Rostock, Germany; 3 Max Planck Institute for Demographic Research, Rostock, Germany

**Keywords:** Population ageing, Old-age dependency ratio, Health indicators, Older workforce

## Abstract

The aim of this study is to answer the question of whether improvements in the health of the elderly in European countries could compensate for population ageing on the supply side of the labour market. We propose a state-of-health-specific (additive) decomposition of the old-age dependency ratio into an old-age healthy dependency ratio and an old-age unhealthy dependency ratio in order to participate in a discussion of the significance of changes in population health to compensate for the ageing of the labour force. Applying the proposed indicators to the Eurostat’s population projection for the years 2010–2050, and assuming there will be equal improvements in life expectancy and healthy life expectancy at birth, we discuss various scenarios concerning future of the European labour force. While improvements in population health are anticipated during the years 2010–2050, the growth in the number of elderly people in Europe may be expected to lead to a rise in both healthy and unhealthy dependency ratios. The healthy dependency ratio is, however, projected to make up the greater part of the old-age dependency ratio. In the European countries in 2006, the value of the old-age dependency ratio was 25. But in the year 2050, with a positive migration balance over the years 2010–2050, there would be 18 elderly people in poor health plus 34 in good health per 100 people in the current working age range of 15–64. In the scenarios developed in this study, we demonstrate that improvements in health and progress in preventing disability will not, by themselves, compensate for the ageing of the workforce. However, coupled with a positive migration balance, at the level and with the age structure assumed in the Eurostat’s population projections, these developments could ease the effect of population ageing on the supply side of the European labour market.

## Introduction

Europe is growing older. In 2010, there were about four people of working age (ages 15–64) for each person of retirement age and above (ages 65+); but in 2050, the ratio is expected to be less than two to one (Eurostat 2011a). There is no universal answer to the question of whether fertility or mortality changes in the past have been the main driving force for the observed population ageing. Dinkel’s (2008) figures suggest that the decline in fertility has further contributed to the ageing of the West German population in recent decades. For other countries—such as France, Italy, Sweden, and the United States—it has been estimated that survival improvements were either equally important or were even the major determinant for population ageing (Caselli and Vallin 1990; Preston et al. 1989). It should be pointed out that Caselli and Vallin (1990) also looked at population projections until 2040 for France and Italy, and concluded that changes in mortality will contribute more to the increase in the elderly population than fertility. For this development to last, it is essential that mortality continues to decrease at advanced ages. Most recent empirical research supports this optimistic view: in Japan, which is characterised by the current value of best-practice life expectancy, the rate of mortality improvements shows no indications of slowing down at ages 80 ages and above, the ages which are now mainly responsible for the increase in life expectancy (Christensen et al. 2009). In fact, even at ages 90 and higher, death rates have been declining at an accelerating pace in recent years (Rau et al. 2008). This trend is not restricted to Japan. Based on changes in smoking patterns, some researchers expect mortality in the United States to decline even faster than in the past (Wang and Preston 2009); Janssen and Kunst (2007) have also asserted that gains in remaining life expectancy at age 80 in seven European countries will be stronger than are typically anticipated when non-smoking-related mortality is taken as the basis for projections.

There is no agreement, however, about the developments in morbidity and disability that will accompany these remarkable improvements in life expectancy. The evidence concerning recent trends in morbidity and disability among the elderly is also mixed (Jagger 2000; European Commission 2010). With poor health being the most important determinant of exit from paid employment in Europe (Burdorf et al. 2005; Kalwij and Vermeulen 2005, 2008; Schuring et al. 2007), the potential growth in the number of the elderly who are still in good health raises the question of whether potential improvements in health and disability could compensate for the ageing process on the labour supply side of the market. The above question cannot be answered, however, with traditional measures of population ageing based solely on chronological age (for example, the old-age dependency ratio). Because they are based on a fixed definition of old age, and disregard developments in the age of the onset of morbidity and disability, these measures are not suitable for answering questions concerning changes in population health and their consequences. Sanderson and Scherbov (2010) were the first, to our knowledge, to propose an alternative measure of ageing that included information on population health. Called the adult disability dependency ratio (ADDR), this measure is constructed as the ratio of the number of adults who are at least 20 years old *with disabilities* to the number of adults who are at least 20 years old *without disabilities*.

Most researchers, journalists and policy makers are familiar with the old-age dependency ratio, as it is still the canonical tool for quantifying population ageing. This is why we chose it as the starting point for our contribution, despite being aware of its aforementioned deficiencies. In our paper, we introduce an alternative approach to including population health in the debate on the consequences of population ageing by decomposing the old-age dependency ratio into two additive indicators: the *old-age unhealthy dependency ratio* and the *old-age healthy dependency ratio*. The traditional old-age dependency ratio represents the weight of the number of old people in relation to the population of working age. Our new indicators distinguish between a) the weight of those who are in good health and could potentially remain in the labour force; and b) those who are disabled or have chronic conditions, and are therefore less likely to work, and are more likely to require financial and other support.

Besides proposing an alternative measure of population ageing, the aim of this work is to apply this indicator to study the consequences of population ageing and the concurrent improvements in health in selected European countries in the years 2010–2050.

## Methods

### Data

In our study, poor health is defined as the prevalence of activity limitations due to health problems. Information on the prevalence of activity limitations by age in 2006 in selected European countries was obtained from the website of the European Health Expectancy Monitoring Unit (2011). These cross-national data originated from the second wave of the Survey of Health, Ageing and Retirement in Europe (SHARE). The sample consisted of over 33 thousand individuals aged 50 years and older in 13 countries. The second wave of the study has been selected because it had a higher number of participating countries and lower non-response rates than the first wave. Participation in the second wave of the study was not dependent on the participation to the first wave as, according to the information provided at the website of the SHARE project, additional to the “respondents from the first wave, a “refresher” sample was also drawn in all first wave countries except Austria and the Flemish part of Belgium.” The non-response rates for the question concerning self-assessed limitations in daily activities were under 0.5 percentage points for all countries, with the exception of France (above 1.5 %) and Switzerland (0.55 %) (Christelis 2008). For additional information on the survey, consult www.share-project.org.

The measurement of activity limitations in this study was based on answers to the following question: “For the past 6 months at least, to what extent have you been limited because of a health problem in activities people usually do?” Those respondents with *severe limitations* and those who were *limited, but not severely* were grouped into single category; thus, respondents with any level of activity limitation were classified as having a disability. The publicly available data report the prevalence of activity limitations in the non-institutionalised population by sex and age in single age groups between 50 and 84, and ending with the open age group 85+. SHARE respondents were limited to the non-institutionalised population. Thus, by not including people in institutions in our study, we underestimate the existing prevalence of activity limitations. However, we can expect this underestimation to be negligible: Sanderson and Scherbov (2010) have shown that including people in nursing homes, with the assumption that the prevalence of disability among this group is 100 %, does not significantly change the value of the *adult disability dependency ratios*.

Thirteen countries were selected for the study based on the availability of data from the second wave of the SHARE survey: Austria, Belgium, Czech Republic, Denmark, France, Germany, Greece, Italy, the Netherlands, Poland, Spain, Sweden and Switzerland. The corresponding population data by age and sex, as of 1 January 2006, were obtained from Eurostat’s Population Database (Eurostat 2011b). Population projections for the selected years between 2010 and 2055 have been derived from Eurostat’s Population Projections (Eurostat 2011a). These projections are based on the assumption that fertility and mortality levels across the countries will converge to those of the “forerunners” by the year 2150. We investigate two scenarios with respect to international migration: the first scenario assumes that there will be zero net migration; while the second scenario allows for migration flows, and assumes that net migration will converge to zero in the same year as the vital events.

## Methods

The old-age dependency ratio is estimated as 100 times the size of the population ages 65+ relative to the total working-age population (ages 15–64). Separating those ages 65+ into healthy and unhealthy groups by applying the corresponding activity limitation rates, the value of this ratio can be decomposed into the old-age unhealthy dependency ratio and the old-age healthy dependency ratio:

$$ \frac{{{N_{{65 + }}}}}{{{N_{{15 - 64}}}}} = \frac{{{N_{{Healthy,65 + }}}}}{{{N_{{15 - 64}}}}} + \frac{{{N_{{Unhealthy,65 + }}}}}{{{N_{{15 - 64}}}}} $$

Assumptions had to be made concerning the future development of the prevalence of activity limitations. In principle, we could have extrapolated trends from the two existing SHARE surveys. We did not pursue this idea. Since the two surveys are only two years apart, any long-term trends obtained from them would be questionable. Instead, we projected future disability rates in the selected European countries based on the idea of the compression of morbidity (Fries 1980, 1989). While two other major hypotheses concerning the development of ill health accompanying the evolution of life expectancy have been proposed—namely, the expansion of morbidity (Gruenberg 1977; Olshansky et al. 1991) and of dynamic equilibrium (Manton 1982)—we chose to follow the scenario of the compression of morbidity. The optimistic scenario was selected in this exercise because it is generally expected that even the improvements in health and disability anticipated in the most optimistic scenario would not compensate for the ageing process on the labour supply side of the market.

We adopted Fries’ “compression of morbidity” theory, which posits that “the end of the period of adult vigor will come later than it used to” (Fries 1980, p.133); i.e., that the proportion of life spent in poor health will be smaller. In contrast to Fries (especially 1989, p. 208), we achieved this not by assuming fewer years of poor health and constant life expectancy, but by setting the years in poor health at a constant number combined with increasing life expectancy (at age 65).

For computational reasons, we further assumed that the age pattern of activity limitations will not change over the projection period. Healthy life expectancy was defined using the Sullivan method (Sullivan 1971).

In the compression of morbidity scenario, we assumed that, between year t and t+n, absolute improvements in life expectancy at 65 years of age equal improvements in healthy life expectancy at the same age. As a result, unhealthy life expectancy does not change (∆*e*_Unhealthy,65_ = 0) and

$$ {e_{{Unhealthy,65}}}(t) = {e_{{Unhealthy,65}}}\left( {t + n} \right) = \frac{{\sum\nolimits_{{x = 65}}^{{ + \infty }} {{p_x}\left( {t + n} \right){L_x}\left( {t + n} \right)} }}{{{l_{{65}}}\left( {t + n} \right)}}, $$

where *p*_*x*_(*t*+*n*) denotes the proportion of those with activity limitations at age *x* in year *t*+*n* and *L*_*x*_, *l*_*x*_ are standard life table functions.

Following the assumption that the age pattern of activity limitations (*k*_*x*_) does not change between *t* and *t*+*n* then ${p_x}\left( {t + n} \right) = p\left( {t + n} \right) \cdot {k_x}\left( {t + n} \right) = p\left( {t + n} \right) \cdot {k_x}(t)$. As *p*_*x*_(*t*+*n*) is independent of age (*x*) then

$$ {e_{{Unhealthy,65}}}\left( {t + n} \right) = p\left( {t + n} \right)\frac{{\sum\nolimits_{{x = 65}}^{{ + \infty }} {{k_x}(t){L_x}\left( {t + n} \right)} }}{{{l_{{65}}}\left( {t + n} \right)}}. $$

Knowing the empirical distribution of *L*_*x*_(*t*) and *L*_*x*_(*t*+*n*), as well as *k*_*x*_(*t*), only the value of *p*(*t*+*n*) is needed for knowing the distribution of activity limitations in year t+*n*, and it can be estimated as

$$ p\left( {t + n} \right) = \frac{{{e_{{unhealthy,65}}}\left( {t + n} \right)}}{{\frac{{\sum\nolimits_{{x = 65}}^{{ + \infty }} {{k_x}(t){L_x}\left( {t + n} \right)} }}{{{l_{{65}}}\left( {t + n} \right)}}}} = \frac{{{e_{{unhealthy,65}}}(t)}}{{\frac{{\sum\nolimits_{{x = 65}}^{{ + \infty }} {{k_x}(t){L_x}\left( {t + n} \right)} }}{{{l_{{65}}}\left( {t + n} \right)}}}} $$

As the available data from Eurostat (2011a) report the number of survivors on 1 January every 5 years between 2010 and 2060 in 5-year age groups between the ages of 65 and 80, we approximate the number of person-years lived between age *x* and *x* +5 in the calendar year *t* by

$$ {}_5{L_x} = 5{l_{{x + 5}}}\left( {t + 5} \right) + {}_5{a_x}\left[ {{l_x}(t) - {l_{{x + 5}}}\left( {t + 5} \right)} \right] $$

where ${}_5{a_x}$ stands for the mean number of person-years lived between t and *t*+5 by those dying in the interval and equals 2.5. In the last open-age interval, the value of *Lx* was approximated by: ${L_{{85 + }}}(t) = {a_{{85 + }}} \cdot {l_{{85 + }}}$, where the value of *a*_85+_ equaled 8.17, which was the highest value of life expectancy at age 85 in 2005–2009; namely, that of Japanese women (Human Mortality Database 2011).

If the prevalence of activity limitations in an age group was one, the fraction of the population with no limitations, and, therefore, also the value of *k*_*x*_ (*t*), would be zero. In this case, we imputed a value of 0.001 for the fraction of those with no activity limitations to allow for improvements in disability prevalence over time in this age group to be calculated according to the proposed estimation method.

## Results

Table 1 gives an overview of the extent and variability of the prevalence of activity limitations at ages 65 and higher in selected European countries in 2006. The prevalence of activity limitations and the age-standardised values are presented in the first two columns. The standard population age structure in this exercise was that of all the countries under study pooled together. In addition to showing the old-age dependency ratio (ODR) in selected European countries, the last three columns of Table 1 present its decomposition into the additive components: the old-age unhealthy dependency ratio (UnHODR) and the old-age healthy dependency ratio (HODR); when summed up, the last two numbers are equal to the value of the total old-age dependency ratio.

**Table 1:** Prevalence of activity limitations, prevalence of activity limitations with the standard European population structure (standardised), old-age dependency ratio (ODR), old-age healthy dependency ratio (HODR) and old-age unhealthy dependency ratio (UnHODR) in selected European countries in 2006

Country	Prevalence of activity limitations (in %) in 2006	Standardised	ODR	HODR	UnHODR
Austria	61	61	24	9	14
Belgium	51	50	26	13	13
Czech Republic	70	71	20	6	14
Denmark	47	46	23	12	11
France	50	49	25	12	13
Germany	60	62	28	11	17
Greece	42	44	27	16	12
Italy	58	57	29	12	17
The Netherlands	54	54	21	10	11
Poland	77	77	18	4	14
Spain	53	53	24	11	13
Sweden	53	52	26	12	14
Switzerland	40	40	23	14	9
					
All	57	55	25	11	14

Authors’ calculations based on Eurostat (2011b) and European Health Expectancy Monitoring Unit (2011)

In 2006, about 57 % of the population ages 65 and older in Europe were living with activity limitations due to health problems. With large variations between the countries, ranging from 40 % in Switzerland to 77 % in Poland, the pattern is far from uniform, however. The differences between countries persist even when the ratios are age-standardised. This means that the variations between the countries in the prevalence of activity limitations result from differences in age-specific morbidity, and not from the age distribution of the populations under study.

In Europe in 2006, there were about 25 people ages 65+ per 100 persons of working age (ages 15–64) . This number varied by country, ranging from 18 in Poland to 29 in Italy. With the high prevalence of activity limitations in the European countries, the value of the old-age unhealthy dependency ratio was higher than the corresponding figure for the healthy dependency ratio. When the 2006 figures for the selected European countries were combined, there were about 14 older people with activity limitations due to poor health per 100 population of working age. The lowest value of the UnHODR, nine, was observed in Switzerland; while the highest value, 17, was found in the Italian and German populations. The old-age healthy dependency ratio, which is the number of people aged 65+ in good health per 100 people of working age, varied between four in Poland and 16 in Greece, with an average value of 11 for all of the countries under study.

In both versions of Eurostat’s population projections for the years 2010–2050, assuming no international migration (Table 2) and allowing for international migration flows (Table 3), the process of population ageing results in a doubling of the old-age dependency ratios in European countries over the studied period. It also appears that, in the absence of other changes, migration flows into European countries in the years 2010–2050, at the level assumed in the Eurostat projection, would do relatively little to relieve the pressure on the social security systems resulting from the ageing population: in 2030, the old-age dependency ratio in Europe would decline from 43 in the projection with no migration to 40 in the projection assuming a positive migration balance. In 2050, the corresponding numbers would be 61 and 52.

**Table 2:** Old-age dependency ratio (ODR), old-age healthy dependency ratio (HODR) and old-age unhealthy dependency ratio (UnHODR) in selected European countries. Projection assuming zero migration, years: 2010, 2030 and 2050

Country	ODR			HODR			UnHODR		
	2010	2030	2050	2010	2030	2050	2010	2030	2050
Austria	26	43	65	13	24	40	13	20	25
Belgium	26	41	51	12	18	24	14	23	27
Czech Republic	22	37	60	8	20	37	14	17	23
Denmark	25	39	47	14	25	33	11	14	13
France	26	41	47	12	19	24	13	21	23
Germany	31	50	67	16	27	41	15	23	27
Greece	28	42	69	16	23	41	13	19	28
Italy	31	47	74	15	22	41	16	25	33
Netherlands	23	42	51	11	23	32	12	19	19
Poland	19	35	55	6	13	20	13	23	34
Spain	25	39	76	12	18	40	12	21	36
Sweden	28	41	48	13	23	31	15	18	17
Switzerland	25	45	66	15	29	45	10	16	21
									
All	27	43	61	13	21	33	14	22	28

Authors’ calculations based on Eurostat (2011a, b) and European Health Expectancy Monitoring Unit (2011)

**Table 3:** Old-age dependency ratio (ODR), old-age healthy dependency ratio (HODR) and old-age unhealthy dependency ratio (UnHODR) in selected European countries. Projection including migration flows, years: 2010, 2030 and 2050

Country	ODR			HODR			UnHODR		
	2010	2030	2050	2010	2030	2050	2010	2030	2050
Austria	26	39	49	13	21	30	13	18	19
Belgium	26	37	42	12	16	20	14	21	22
Czech	22	34	50	8	18	31	14	16	19
Denmark	25	37	42	14	24	30	11	13	12
France	26	39	45	12	19	23	13	20	22
Germany	31	47	58	16	25	35	15	22	23
Greece	28	38	57	16	21	34	13	17	23
Italy	31	41	56	15	19	31	16	22	25
Netherlands	23	40	47	11	22	29	12	18	18
Poland	19	35	53	6	13	20	13	23	33
Spain	25	36	57	12	17	30	12	19	27
Sweden	28	37	42	13	21	27	15	16	15
Switzerland	25	38	51	15	24	35	10	14	16
									
All	27	40	52	13	20	34	14	20	18

Authors’ calculations based on Eurostat (2011a, b) and European Health Expectancy Monitoring Unit (2011)

Despite the large variation between the countries, the projected values of the old-age dependency may be expected to rise over the years in all of the countries under study. Germany could be considered the forerunner in the ageing process: in 2030, there will be only about two people of working age for each person aged 65+; and, assuming zero migration, the old-age dependency ratio can be expected to rise to 67 by 2050 (with a positive migration balance, it will be 58). In the Southern European countries in 2050, the situation is projected to be even more severe, with about four people of working age per three retirees in the zero-migration scenario (with an ODR of between 69 in Greece and 76 in Spain).

Even with improvements in population health that ensure that the increase in healthy life expectancy matches the rise in life expectancy over the period 2010–2050, the growing number of elderly people may be expected to lead to an increase in both the healthy and the unhealthy dependency ratios. The unhealthy dependency ratio will make up the larger part of the old-age dependency ratio in Europe. The value of UnHODR is projected to rise by 43 % between 2010 and 2030 (57 % in the no-migration projection); as a result, in 2030 there will be 100 people between the ages of 15 and 64 to support about 20 (22 in the no-migration projection) elderly people in poor health in Europe. At the same time, these 100 working-age people will financially support 20 retirees ages 65+ (21 in the no-migration projection) with no activity limitations. Only in five out of the 13 selected countries (Belgium, France, Italy, Poland and Spain) is the HODR expected to be lower than the corresponding value of the unhealthy ratio. In other words, in the majority of the countries under study, at least half of the financial burden of the elderly on the working-age population in 2030 will come from the portion of the elderly population with no activity limitations.

Our estimations with a zero-migration balance indicate that there will be 28 elderly in poor health and 33 still in good health per 100 people in the current working age range of 15–64 in Europe in the year 2050. In the positive migration balance scenario of the projection, the above numbers will be 18 and 34, respectively. Thus, as in 2030, in 2050 over 50 % of the old-age dependency ratio will be made up of the healthy old-age dependency ratio. In 2050, the value of the old-age healthy dependency ratio will be lower than the unhealthy ratio in Belgium and Poland only. Thus, with improvements in population health in the second period under study, in almost every European country a major part of the financial burden associated with the elderly may be expected to come from supporting those with no activity limitations preventing them from working.

In the most optimistic of the three possible scenarios of future developments of morbidity and disability, and with the zero-migration assumption, in the year 2050 the value of the unhealthy old-age dependency ratio in Europe will be higher than the present (2010) value of the old-age dependency ratio. This means that improved health among the elderly will not by itself compensate for the process of population ageing. In the scenario that includes migration flows, however, the value of unhealthy old-age dependency ratio will be 10 points lower than in the former scenario in 2050. This would be accompanied by a difference of only one point in the value of healthy old-age dependency ratio between the two scenarios. Thus, in 2050 in the positive migration scenario, 65 % of the old-age dependency ratio may be expected to be composed of the healthy ratio, compared to 54 % in the zero-migration balance projection. This difference in the distribution of the old-age dependency ratio between the old-age healthy and unhealthy dependency ratios in the two scenarios is a result of differences in the age composition of the population in the two population projections as disability rates in all age-groups in both scenarios are identical. In the projection that allows for migration flows, it is assumed that at the moment they move into one of the European countries, future migrants will be of younger working ages. Although by the year 2050 many of the migrants will have reached the retirement age of 65, they will still be younger among the old-age groups. Hence, in the projection allowing for migration flows, the proportion of those in the younger elderly groups will be higher as compared to the alternative scenario with zero migration. With younger elderly characterised by lower disability rates as compared to older age-groups, this compositional difference will result in a lower proportion of those with disabilities in the scenario allowing for migration flows than in the scenario with zero migration.

We conclude the presented analysis by observing that the future of the European labour market looks very different if we combine the projected improvements in the health of the elderly with a positive international migration balance. In the worst-case scenario, with the strong theoretical assumptions that the entire population aged 65+ are inactive and there is zero-migration balance between 2006 and 2050, there would be 61 retirees per 100 persons of working age in the selected 13 European countries in 2050. On the other hand, the optimistic scenario applied in this study assumes positive migration flows over the years studied, and that only those with activity limitations at older ages will be out of the labour market. Our interpretations here are based on the additional strong assumption that all those with no activity limitations at older ages will remain in the labour market. In this optimistic scenario, the number of retirees would be 18 per 100 persons at working ages; that is, less than 30 % of the corresponding value in the scenario we labelled as pessimistic. Taking into account the potential pathways of single countries, the worst-case scenario would result, for example, in 100 working-age people per 76 retirees in Spain (in 2050 the highest value of ODD in the zero-migration projection is 76 in Spain). Meanwhile, in the optimistic scenario for the same country, 100 people of working age will be financially supporting only 27 retirees in poor health. We conclude that, although neither a positive migration balance nor the improved health of the elderly can compensate for the process of population ageing, the combination of the two will make a big difference for the future of the European labour market in the 40 years to come.

## Discussion

We decomposed the old-age dependency ratio into the old-age unhealthy dependency ratio and old-age healthy dependency ratio in order to participate in the discussion about the importance of improvements in the health of the elderly for the ongoing process of labour force ageing. Based on Eurostat’s population projections, and assuming equal improvements in life expectancy and healthy life expectancy at birth, we estimated values of the three indicators in selected European countries in 2010–2050. In our scenarios, we demonstrated that improvements in health and disability will not compensate for the ageing process on the supply side of the labour market, even though growing numbers of the elderly will still be in good health. It should be acknowledged, however, that these improvements could notably ease the burden of the elderly on the working age population, in particular in the scenario with a positive migration balance. The effect of the young age composition of migrants could have a significant effect on the value of the old-age dependency ratio by the year 2050. Compared to the zero-migration scenario, the lower value of the old-age dependency ratio would translate directly into a change in the old-age unhealthy dependency ratio. With the strong assumption that the working-age population will be supporting only the elderly with activity limitations, and that those with no activity limitations will remain in the labour market, the number of retirees per person of working age in 2030 will be 0.2 in the scenario that allows for migration flows. In other words, this value of 0.2 will be 26 % lower than the current value of the old-age dependency ratio in selected European countries. In the same scenario, the corresponding number will be 0.18 in 2050; that is, 33 % lower than the current value of the old-age dependency ratio.

We acknowledge that the study has two major limitations. The first of the limitations is related to the fact that the age range 15–64 is used to define working ages in the old-age dependency ratio. Although 65 is the standard age of eligibility for pension benefits in two-thirds of OECD countries, labour force participation rates tend to drop significantly between the ages of 55 and 64 (compare, for example, Blöndal and Scarpetta 1998; Börsch-Supan et al. 2009; Duval 2003). The average age of retirement is, for instance, currently around 60 in Germany (Deutsche Rentenversicherung 2011). We can expect, however, that with the shortage of labour and the growing financial problems faced by the retirement systems in European countries, the early retirement schemes and transfer programs used as early retirement schemes will be abandoned in the projection period, and the official retirement age will be postponed. For example, a reform that leads to a gradual postponement of the statutory retirement age in Germany (from age 65 today to age 67 in 2030) was passed in 2007 (Wilke 2009). Likewise, due to changes in participation in education, using 15 as the entry age into the labour market is too low today. Even at ages 20–24, only 36.8 % of people are part of the working population (*Erwerbstätige*) in Germany (Wingerter 2011). Being aware of those shortcomings concerning the definition of working age, we decided to use the standard definition of the old-age dependency ratio, as any change in the discussed ages would be equally questionable.

The second major limitation is the uncertainty about whether self-rated health is a good indicator of health that should to be linked to retirement behaviour. Based on the justification hypothesis, it is possible that people who retire early seek to rationalise their decision by rating their health as poor. However, empirical studies that include both self-rated health and measures of health based on bio-markers have not confirmed the existence of the justification hypothesis (Bond 1989; Kalwij and Vermeulen 2005, 2008).
